# Supplementary material for: Unconscious categorization of sub-millisecond complex images
Source: PLoS One. 2020 Aug 12;15(8):e0236467. doi: 10.1371/journal.pone.0236467 (PMC7423101; doi:10.1371/journal.pone.0236467)
Supplement: S1 File — (DOCX) [file pone.0236467.s001.docx]

Supplementary Material

Experimental setup

Our tachistoscope is an adapted version of the design of Sperdin et al. (1). The functional principle of both theirs and our tachistoscope is that two LCD screens are used, one placed upright vertically and the other horizontally aligned to the top of the other screen (Fig S1.A). Both screens are fixed in a rigid frame made from aluminium extruded beam (Fig S1.B). The frame is covered internally and externally by black matte plexiglass to void light reflection (Fig S1.C).

**Fig S1.** **Illustration of our tachistoscope design.** a) A semi permeable mirror is positioned between two monitors arranged in a 90° configuration. One of the monitor’s light is reflected (screen 1), while the second’s passes through the mirror (screen 2). b) The screens and mirror are supported by a rigid structure in aluminium. c) The complete device is covered with black plexiglass. An optional front plate with a small aperture can be added to ensure optimal head position and minimal room light interference.

A diagonally placed semi-permeable mirror in between the two screens allows the light to pass through from the back screen while it reflects light emitted from the top screen (Fig S1.A). Both screens therefore appear to the participant to be superimposed on each other if the backlight of both screens are turned on simultaneously. By controlling the backlight of each screen separately we can precisely control which screen is visible to the subject at each timepoint with a precision of 2 microseconds (+-1), which allows such brief stimulus durations. The difference with the design of Sperdin et al. (1) is that the screens’ backlights are not directly controlled by the computer using a parallel port, but with a dedicated microcontroller (ATmega328 AVR) instead. The precise timing is sent by the computer to the microcontroller via serial communication (USB 2.0).

Using a dedicated microcontroller offers two main advantages compared to the parallel port:

- First, parallel ports are becoming very uncommon on computers (often requiring outdated computers or very expensive add-on cards).
- Second, the microcontroller is more precise and more consistent than the parallel port. Indeed, as its only task is to control the blacklight, the microcontroller is not affected by the load on the computer. It can also be used by many different computers without changing its performance. In comparison, using a parallel port was difficult to control as it required to prioritize the backlight command by providing it high access in software (MATLAB). It was also directly affected by the type of computer.

The drawback of using a microcontroller is that “when” the stimulus is presented is affected by the time it takes for the computer to send the information (the serial communication). This time has an order of magnitude of a few milliseconds and is not constant (2). Consequently, while the stimulus duration is perfectly controlled, the moment of its exact appearance can vary with hardware and software load.

Once the microcontroller receives the instruction via serial communication, it then switches the backlight to present the stimulus for the required time. The required stimulus duration can vary between 10 microseconds to 10 seconds. A temporal precision of +- 1 microsecond was achieved for stimulus under 16 milliseconds and of +- 20 microseconds for stimulus between 16 milliseconds and 10 seconds. An additional signal output allowed to record screens status (stimulus duration) to synchronise it with various recording devices such as EEG.

Other material

The LCD screens used where Philipps 223V5LHSB2 with a resolution of 1920x1080 pixels for a size of 476.6mm in width and 268.11mm in height. Therefore, the pixel pitch was of 0.248x0.248mm. In addition, the opercula-screen distance was of 360mm (between the middle of the screen to the human nose contact with the tachistoscope opercula). When not performing a “stimulus” only one LCD screen was visible and behave like a typical LCD screen with a response time of 5ms and with a 60Hz refresh rate.

The semi-permeable mirror used was a Pilkington **MirroView™**50/50 glass of 418 x 504 mm. The glass had a 6mm thickness and was toughened for robustness. Its high rigidity avoided any image deformation due to mirror deflection caused by gravity (a problem that appeared in the prototype with an acrylic mirror).

The backlight was powered by an independent power supply (36V 108Watt) with 20 Ohms rheostat in series to dim the screen luminosity. Indeed, the semi-permeable mirror was not perfectly “50:50” resulting in one of the two screens being more luminous than the other. Using a rheostat allowed to decrease the voltage of the more luminous screen to match the one with lower luminosity. It also allowed to correct for screen disparities as two screens are never exactly the same. Consequently, the overall luminosity of the tachistoscope was less than half the luminosity of the screen (see in measurement for exact value).

Measurements

To measure the tachistoscope temporal response, we used the same sensor as Sperlin et al. (1): two photodiodes (Thorlabs PDA36A-EC) set to a gain of 30DB (one for each monitor) and equipped with Pentax 50mm-F1.4 lens and a spacer. Both diodes where placed directly in contact with the monitors in such a way that they would not be affected by the light of the other monitor. Data was collected using a digital oscilloscope (ADS1002CAL). The measurements showed that the tachistoscope exhibits an exceptional temporal precision (Fig S2).

**Fig S2.** **The tachistoscope has a high temporal precision.** Here are examples of stimuli ranging from *20* μ*s* to *1000* μ*s* (1*ms*). The luminosity of each screen is measured separately and we observe that the tachistoscope is precise to 1μ*s.* We also observe a small transitional phase of 2μ*s* caused by the switch of monitors.

In addition, we also measured the combined luminance (as perceived by the participants) of both screens when presenting a stimulus. Indeed, when a stimulus is presented a “switch occurs” where one screen is turning off while the other is turning on. During this transition phase, the light of both screens is combined. It that can cause either a drop of luminosity or a burst of luminosity. We therefore tried to limit this peak in such a way that the transition seems flawless. However, because the rising and falling curves for the screen luminosity are not symmetrical, a perfect flawless transition is impossible. In such case, it is preferable to have a transition with a small loss in combined luminosity (less perceptible than a flash). In all cases, the duration of the transition is around 2 micro seconds (Fig S3), so probably negligible.

**Fig S3. Detailed measurements for a stimulus of 500 micro-seconds.** Switch between the two screens: Resulting luminosity is a combination of the two screens and do not produce a visible variation of luminosity. To display a stimulus, the main screen (screen 1) is powered off while screen 2 is powered on. After the stimuli duration, screen 2 is powered off and screen 1 powered on. The “computed” curve (in black) is a result from addition of screen 1 and screen 2, while the “measured” curve (in green) is obtained experimentally directly on the tachistoscope.

To tune and measure the luminance of the tachistoscope for both screens, we used the Konica LS-150 luminance meter located at the centre of the tachistoscope at 360mm from the screen / virtual screen and pointing towards it. Using the rheostat, we tuned both screens (see Fig S4) in order to achieve a resulting luminosity (after reflection / transmission through the semi-permeable mirror) of 80 cd/m².

**Fig S4. Tuning of the tachistoscope.** Tuning of the screen luminosity. Because of the mirror asymmetry and the LCD difference, the perceived luminosity of the screen can vary (black). Using a rheostat in series with the screen blacklight, tuning is performed to set both screens at a similar value (blue). The spikes correspond to the switch between the 2 monitors. Calibration was performed on a 500μ*s* stimulus.

References

1. Sperdin HF, Repnow M, Herzog MH, Landis T. An LCD tachistoscope with submillisecond precision. Behav Res Methods. 2013 Dec 1;45(4):1347–57.

2. Korver N. Adequacy of the Universal Serial Bus for real-time systems. :36.
