# Supplementary figures and images for: Unconscious categorization of sub-millisecond complex images
Source: PLoS One. 2020 Aug 12;15(8):e0236467. doi: 10.1371/journal.pone.0236467 (PMC7423101; doi:10.1371/journal.pone.0236467)

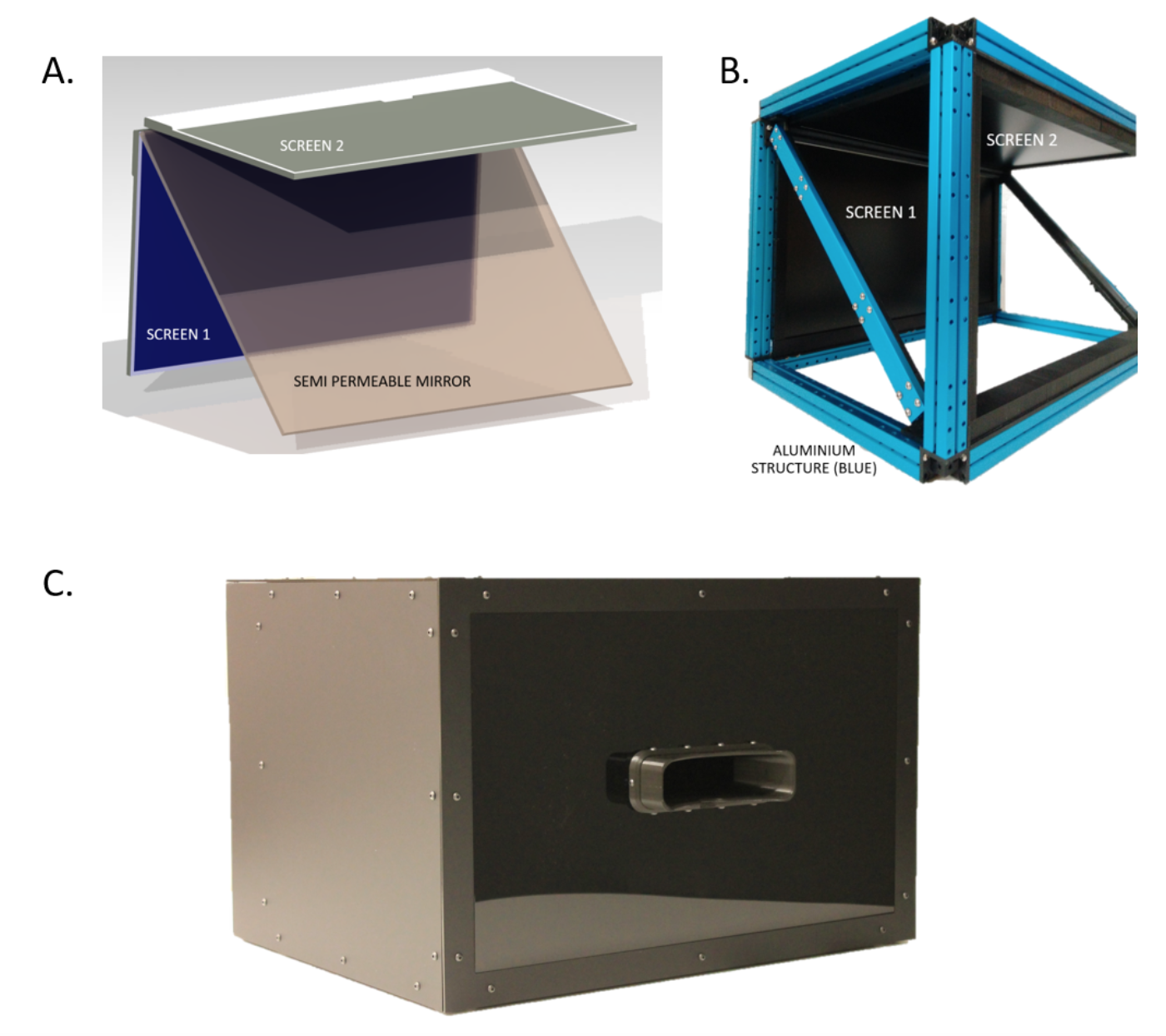

Supplement: S1 Fig — a) A semi permeable mirror is positioned between two monitors arranged in a 90° configuration. One of the monitor’s light is reflected (screen 1), while the second’s passes through the mirror (screen 2). b) The screens and mirror are supported by a rigid structure in aluminium. c) The complete device is covered with black plexiglass. An optional front plate with a small aperture can be added to ensure optimal head position and minimal room light interference. (TIF) [file pone.0236467.s002.tif]

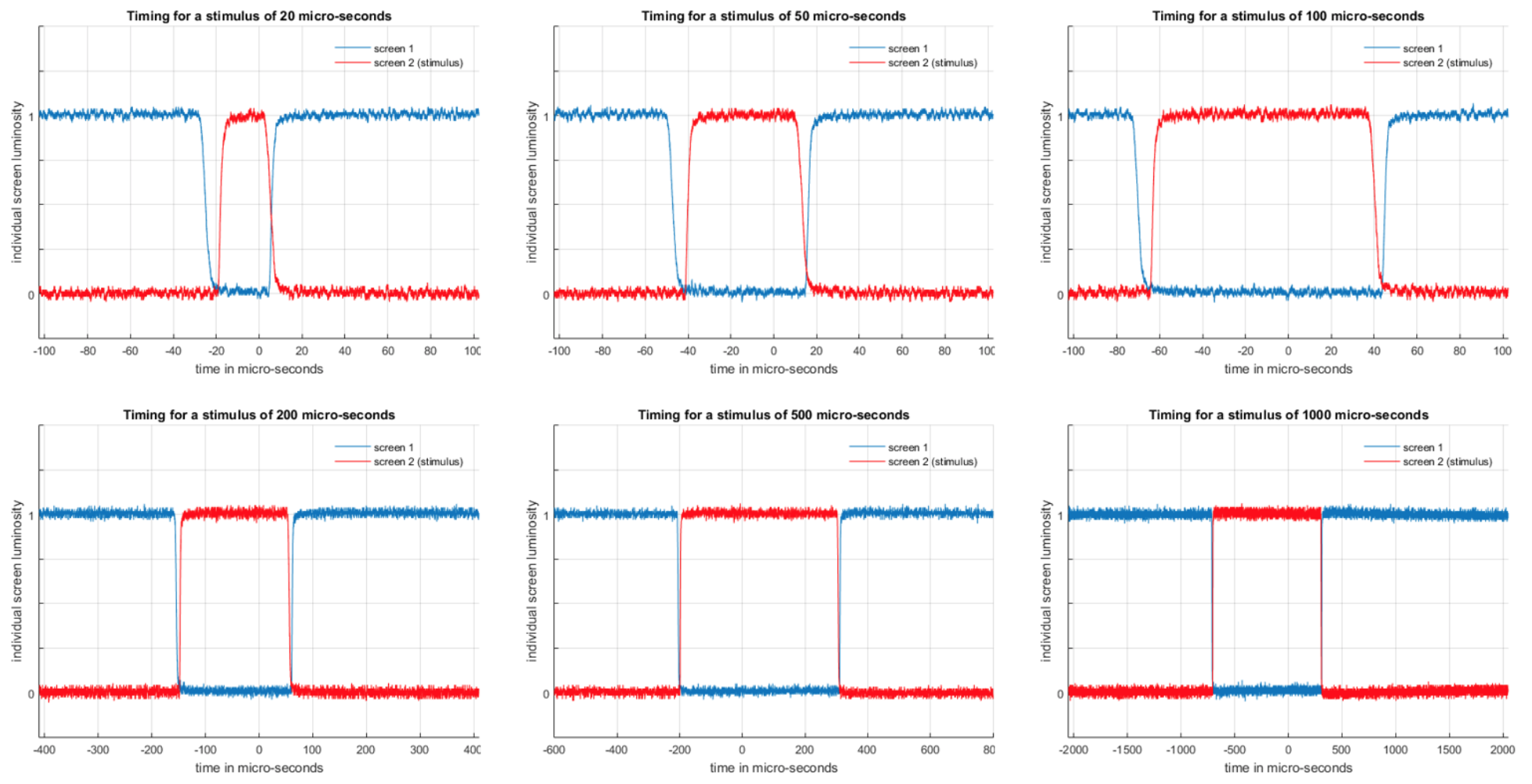

Supplement: S2 Fig — Here are examples of stimuli ranging from 20 μs to 1000 μs (1ms). The luminosity of each screen is measured separately and we observe that the tachistoscope is precise to 1μs. We also observe a small transitional phase of 2μs caused by the switch of monitors. (TIF) [file pone.0236467.s003.tif]

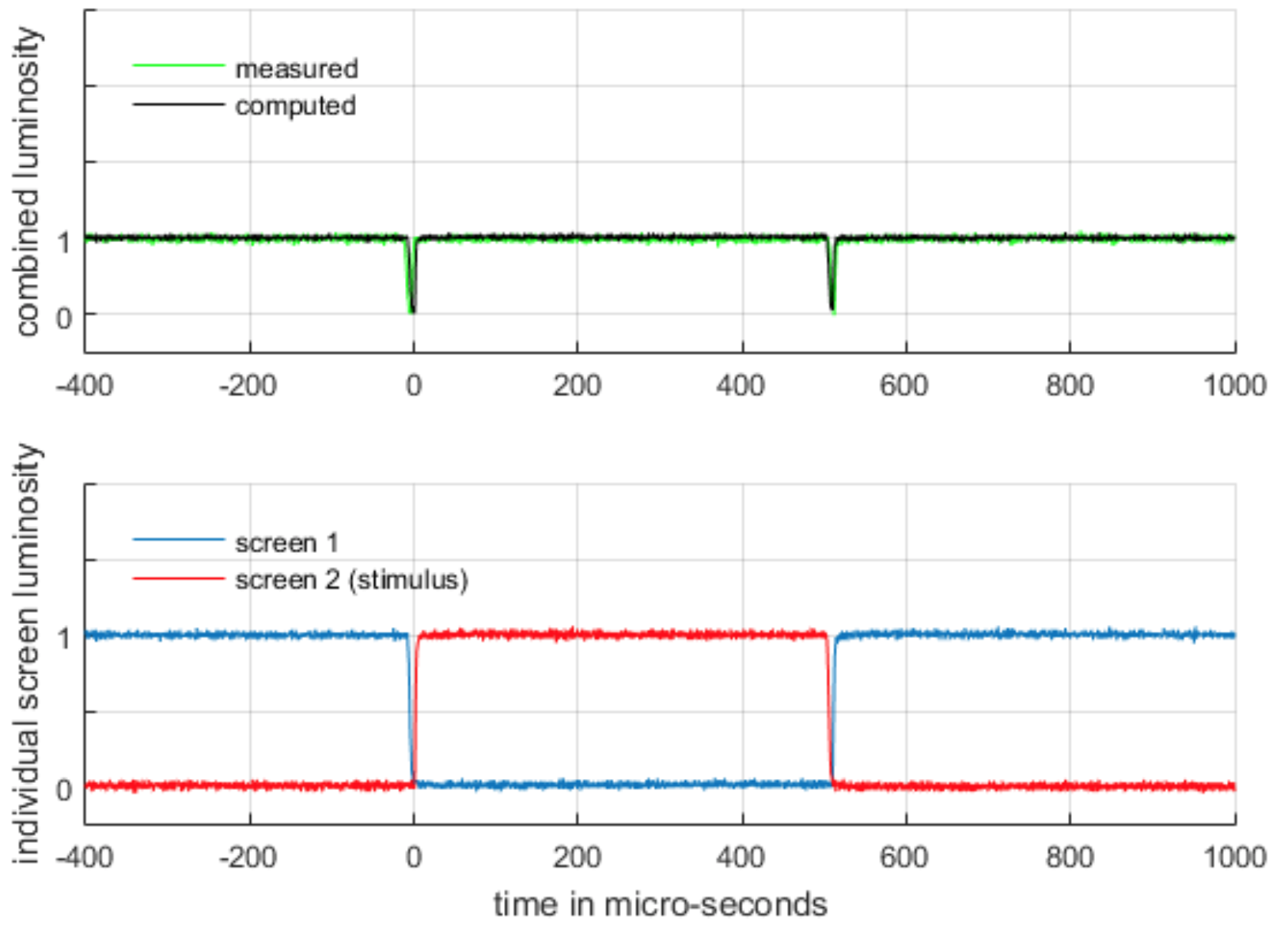

Supplement: S3 Fig — Switch between the two screens: Resulting luminosity is a combination of the two screens and do not produce a visible variation of luminosity. To display a stimulus, the main screen (screen 1) is powered off while screen 2 is powered on. After the stimuli duration, screen 2 is powered off and screen 1 powered on. The “computed” curve (in black) is a result from addition of screen 1 and screen 2, while the “measured” curve (in green) is obtained experimentally directly on the tachistoscope. (TIF) [file pone.0236467.s004.tif]

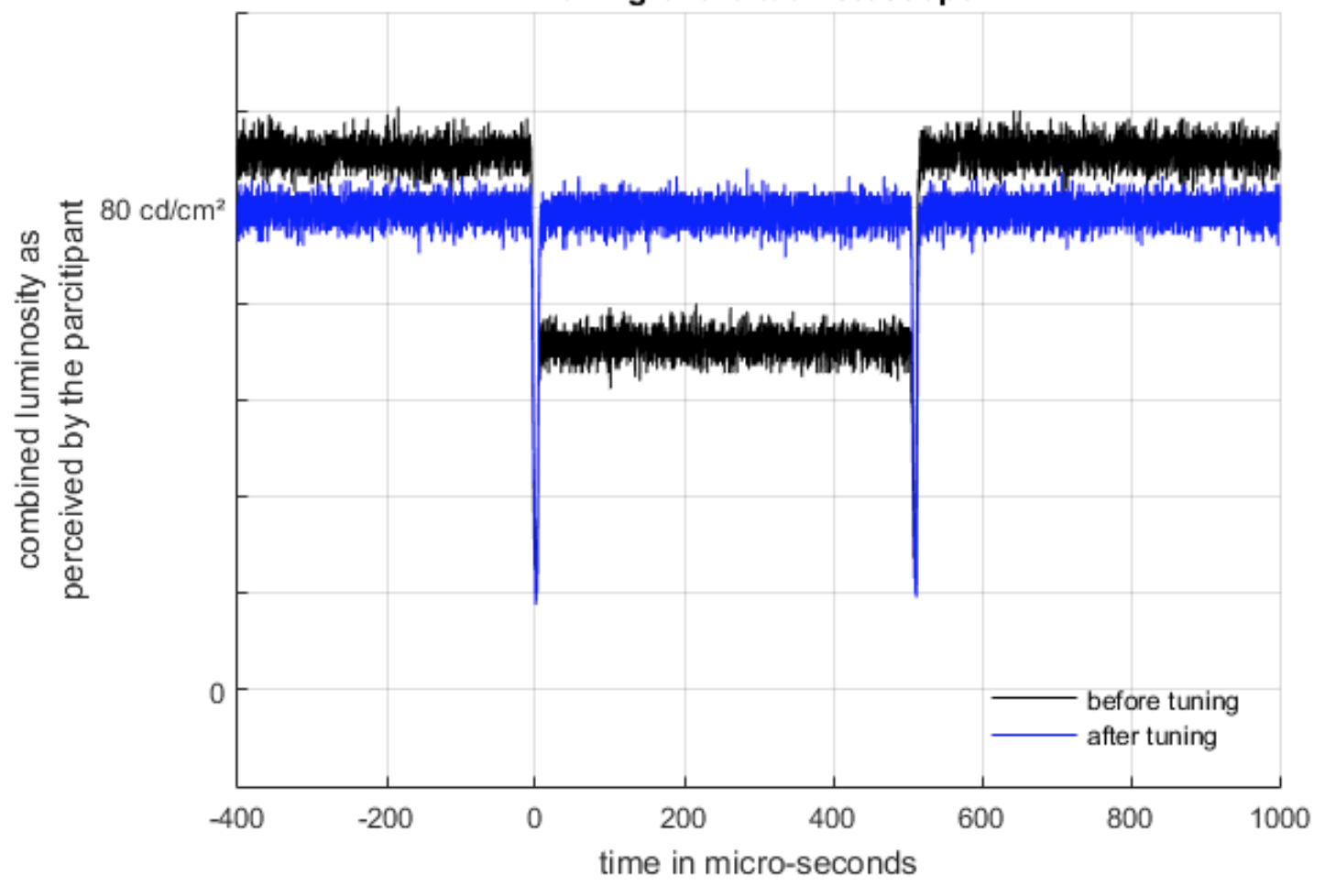

Supplement: S4 Fig — Tuning of the screen luminosity. Because of the mirror asymmetry and the LCD difference, the perceived luminosity of the screen can vary (black). Using a rheostat in series with the screen blacklight, tuning is performed to set both screens at a similar value (blue). The spikes correspond to the switch between the 2 monitors. Calibration was performed on a 500μs stimulus. (TIF) [file pone.0236467.s005.tif]
